# COVID-19 policies and tuberculosis services in private health sectors of India, Indonesia, and Nigeria

**DOI:** 10.1016/j.jctube.2024.100503

**Published:** 2024-12-02

**Authors:** Nathaly Aguilera Vasquez, Charity Oga-Omenka, Vijayashree Yellappa, Bony Wiem Lestari, Angelina Sassi, Surbhi Sheokand, Bolanle Olusola-Faleye, Lavanya Huria, Laura Jane Brubacher, Elaine Baruwa, Bachti Alisjahbana, Madhukar Pai

**Affiliations:** a McGill International TB Centre, Montreal, Canada; b School of Public Health Sciences, University of Waterloo, Waterloo, Canada; c TB PPM Learning Network, Research Institute of McGill University, Canada; d Research Center for Care and Control of Infectious Disease, Universitas Padjadjaran, Bandung, Indonesia; e Department of Public Health, Faculty of Medicine, Universitas Padjadjaran, Bandung, Indonesia; f Global Development Group, Abts Global, Abuja, Nigeria; g Department of Internal Medicine, Hasan Sadikin General Hospital, Bandung, Indonesia; h Department of Epidemiology, Biostatistics and Occupational Health, McGill University, Montreal, Canada

**Keywords:** COVID-19, Private sector, TB healthcare, India, Indonesia, Nigeria

## Abstract

**Introduction:**

The COVID-19 pandemic created unprecedented challenges in the field of global health. Nigeria, Indonesia and India are three high tuberculosis (TB) burden countries with large private health sectors. Both TB and the private health sector faced challenges in these countries because of COVID-19. This study aimed to compare the COVID-19 control measures and policies in the provision of TB care services and gain insights from policymakers on how the pandemic affected the provision of TB services in the private healthcare sector, how each country adapted, and identify lessons learned for health system preparedness.

**Methods:**

Qualitative, in-depth interviews were conducted among a purposive sample of 11 national and sub-national policymakers in each country. Thematic content analysis was conducted on the data collected using an adapted WHO Health Equity Policy Framework.

**Results:**

Results revealed three policy dimensions under costs, access, and quality. Under healthcare costs, policymakers highlighted resource allocation and diversion of TB resources to COVID response, and increased operational costs for private provider. Under healthcare access, key themes included reduced TB case detection due to fear of COVID-19, disrupted diagnostic services, and adaptations such as extended medicine supplies and tele-consultations. Under healthcare quality, themes included compromised TB diagnostic accuracy due to similar respiratory symptoms with COVID-19, and strain on laboratory infrastructure due to competing demands from both diseases. Policymakers across the three countries pointed to the need for strengthening private–public partnerships (PPP) for healthcare service delivery and continued private sector investment to facilitate the continuity of TB care within a pandemic context.

**Conclusion:**

The results of this study provide an overview of the impact of the pandemic from the perspective of private facilities and policymakers in Nigeria, Indonesia and India, which can inform future policy and ways forward in strengthening PPP for healthcare service delivery in high TB burden countries.

## 1 Introduction

On March 11th, 2020, COVID-19 was declared a pandemic by the World Health Organization (WHO). This was a pivotal moment for many countries across the world and led to immediate implementation of infection control measures and lockdowns. Many health providers were challenged with how to offer care in unprecedented conditions. Over three years later, the global health community is now beginning to understand the devastating effects of the pandemic on health service delivery. One area that has been disproportionately affected is tuberculosis (TB) service provision and access due to healthcare provider closures and decreased ability to access care due to lockdown restrictions, among other factors [1], [2], [3].

TB, a disease driven by poverty and disproportionately affecting the most vulnerable populations [4], has long been plagued by systemic inequities across the care cascade [5], [6]. Before the onset of the COVID-19 pandemic, ensuring equitable healthcare had been a particular challenge for global TB control, with significant proportions of infected individuals not accessing care. Several studies within high-burden TB countries have highlighted several challenges with ensuring equity of access, costs and quality in TB care, and advocated for different strategies in TB control [7], [8], [9], [10].

Estimates showed an approximate 18 % decrease in TB case notifications in 2020 compared to 2019 [11]. This improved in 2021, which saw a 5 % increase in TB cases diagnosed and reported [12]. This reflects various challenges encountered in accessing TB care and how service providers adapted throughout the pandemic. Nigeria, Indonesia and India are three of the highest TB burden countries in the world and are among eight countries that accounted for two-thirds of TB cases in 2021. Although India and Indonesia are among five countries that accounted for over 90 % of the reduction in TB case notifications in 2020 compared to 2019, Nigeria reported a 15 % increase in notifications in 2020 compared to 2019 and a further 50 % increase in 2021 compared to 2020. Additionally, all three countries have a large private health sector which accounts for a large proportion of initial care-seeking for TB: 74 % in India, 74 % in Indonesia, and 67 % in Nigeria [13]. The private health sector accounts for an important share of healthcare expenditure in these countries (72 %, 51 %, and 78 %, respectively) [13]. Despite having the majority share of initial care-seeking for TB and healthcare expenditure, the private sector only accounts for a relatively small proportion of TB notifications in the three countries (25 % in India, 18 % in Indonesia, and 12 % in Nigeria) [13].

**Fig. 1:**
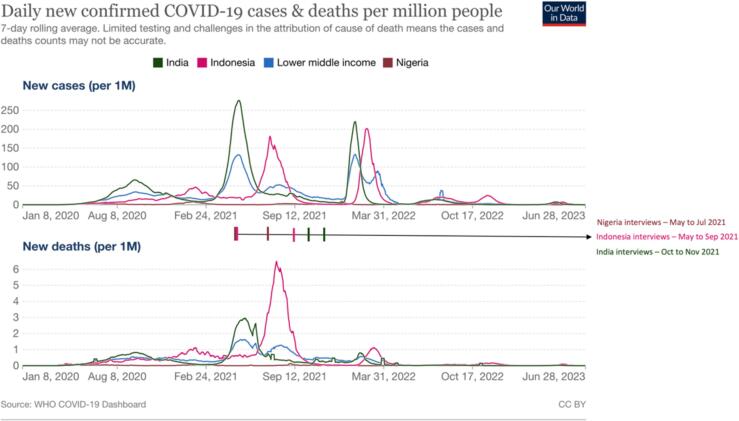
Data collection timeline and COVID-19 infection epi-curves for Nigeria, Indonesia and India with infection trends for lower and middle-income countries. [20].

The COVID-19 pandemic’s impact varied across these three countries − with Indonesia and India documenting large infection waves in 2021 and 2022, while Nigeria’s reported cases remained relatively low compared to other lower and middle-income countries (see Fig. 1 WHO-reported cases by country) [14]. While actual infection rates were likely higher across all countries due to varying testing and reporting capacities, the pattern and timing of waves remains consistent with reported data. All three countries implemented policies to reduce infection and disease transmission during the pandemic. In Nigeria, the government’s previous experience with disease outbreaks such as Ebola enabled the country to be particularly prepared for the COVID-19 pandemic [15]. Preparedness in Nigeria began in late January before the country’s first case of COVID-19 with the inauguration of the National Coronavirus Preparedness Group and, shortly after, the Multisectoral Technical Working Group [15]. Following the first case, the Presidential Task Force was established which implemented the National COVID-19 Multi-Sectoral Pandemic Response Plan, enabling multi-sectoral, coordinated governmental response [15]. Nigeria had an initial lockdown period in March 2020, which was followed by a second lockdown period in December 2020. The first case of COVID-19 in Indonesia was announced on March 2, 2020 [16], [17]. On March 13, 2020, President Joko Widodo established a COVID-19 Task Force, which provided live updates to the public on an official government website. Large scale social restrictions were placed in effect beginning on March 31, 2020 that included closure of schools and workplaces, and restriction of religious activities, activities in public spaces and facilities, social and cultural events, and transportation [6]. These restrictions were superseded in January 2021 by a new set of restriction levels called *Pemberlakuan Pembatasan Kegiatan Masyarakat,* or PPKM, which were dynamically revised several times according to the latest COVID-19 situation in the country. In India, a 21-day lockdown was announced on March 24, 2020 which was extended until October of the same year [18]. Although India’s central government could impose preventative infection control measures such as border measures, lockdowns, curfews, and a ban on mass gatherings, the State governments were charged with the legislation and enforcement of public health measures in their respective states, as well as implementing their own COVID-19 policies [18].

In high TB burden countries, public–private partnerships (PPP) play a crucial role in TB care delivery, as significant proportions of patients seek care in the private sector. All three countries implemented policies that impacted TB care during the pandemic, with their substantial private health sectors significantly affected. While previous research has examined COVID-19′s impact on TB private provider engagement through intermediary non governmental organizations (NGOs) [19], there remains a critical need to understand policy adaptations at national and sub-national levels. This study aimed to compare the COVID-19 control measures and policies through an equity lens, analyse policymakers’ insights on pandemic effects on private sector TB service delivery (focusing on access, costs, and quality), and identify lessons learned for health system preparedness.

## 2 Methods

### 2.1 Study setting

This study formed part of the COVID-19 Effects on TB Services in the Private Sector (COVET) study conducted by the McGill International TB Centre, Georgetown University and the University of Waterloo, evaluating pandemic policy impacts on the private healthcare sector in three high TB burden countries – Nigeria, Indonesia and India [21], [22], [23], [24], [25], [26]. The study examined private sector TB services, including patient pathways, quality of care, and care-related costs, and policy environment changes.

In Nigeria, implementation occured in Kano and Lagos states through the USAID-funded *Sustaining Health Outcomes through the Private Sector* (SHOPS) Plus program implemented by Abt Global, which formed a network of private community health providers trained in appropriate TB screening, diagnosis, and treatment practices [27]. In Indonesia, a partnership with the Universitas Padjadjaran built on their previous *Investigation of Health Services for TB by External Private Providers* (INSTEP) study in 2017–2018 and within the same study area in Bandung, West Java [28], [29]. In India, the TB Public-Private Mix Learning Network (TBPPM LN**)** collaborated with our main partner, Institute of Socio-Economic Research on Development and Democracy (ISERDD) to implement the study, building on previous studies [30], [31].

### 2.2 Data collection

Qualitative, in-depth interviews were conducted virtually between May-November 2021, with each interview lasting 45–60 min. Using maximum variation purposive sampling, we selected 11 national and sub-national policymakers in each country (33 total) based on their direct involvement in TB policy during the pandemic and minimum of two years’ TB program experience. Interviews were conducted and supervised by trained qualitative researchers (VY, BOF and BWL) and research assistants from each country's research institution, who had no prior direct working relationships with participants. Initial contact was established through official TB program channels. Interviews were conducted in English for Nigeria and India, and in Bahasa (translated to English) for Indonesia. All interviews were audio-recorded with permission and transcribed verbatim, with data collection continuing until thematic saturation was achieved (assessed by VY, BWL, CO). No repeat interviews were required. Data analysis combined deductive coding using the WHO Health Equity Policy Framework with inductive coding to identify emerging patterns (conducted by NAV, VY and CO).

Key informant interviews were conducted with mid- and senior-level policymakers from NGOs at national, sub-national, and district levels who were responsible for TB and COVID-19 policies. A standardized interview guide explored policy changes and service adaptations during the pandemic. Ethics approval for this study was provided by the following institutions: the McGill University Health Centre Research Ethics Board (COVID BMGF / 2021–7197 on 19/01/2021), and the Research Ethics Committee of Universitas Padjadjaran, Bandung, Indonesia (No.166/UN6.KEP/EC/2021 on 08/03/2021). In Nigeria, we received approval from the Health Research Ethics Committee of the Lagos State University Teaching Hospital (LREC/06/10/1517 on 19/02/2021) and Kano State Ministry of Health (MoH/Off/797/T.1/2168 on 10/02/2021). In India, we received approval from the Institutional Review Board of the Institute for Socio-Economic Research on Development and Democracy (ISERDD/150221 on 15/02/2021).

### 2.3 Data analysis

**Fig. 2:**
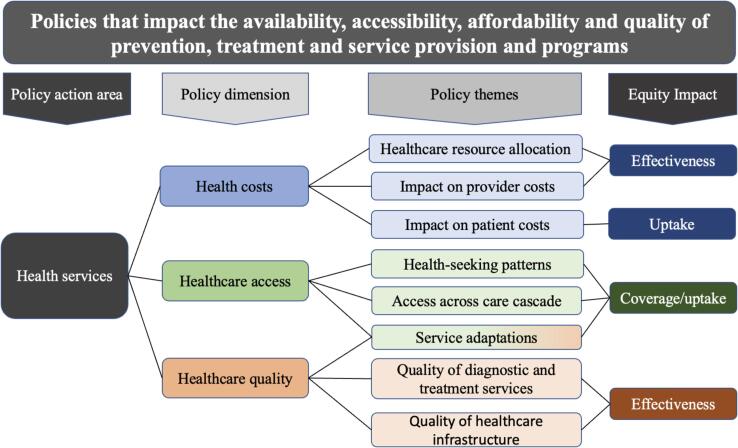
Adapted WHO Health Equity Policy framework on policy changes in TB services during COVID-19 in India, Indonesia and Nigeria.

Transcripts were analyzed using thematic content analysis using Quirkos.​ We adapted the WHO health equity policy framework [32] to look at the impact of COVID-related policy changes on private sector healthcare (Fig. 2). The framework focuses on the policy action areas of health services, with policy dimensions of health costs, healthcare access, and healthcare quality. We identified policy indicators and indicator types. Themes were broken down according to each area and expanded upon using the findings from our study.

## 3 Results

A total of 33 policymakers were interviewed: 11 in each country. Of these, 22 worked at the government level, 10 worked with non-profit organizations, and one worked in an academic institution. Additionally, 14 key informants worked at the national-level, 12 at the state- or provincial-level, and seven at the city-level or district-level. Additional key informant information can be found in Table 1.

**Table 1:** Characteristics of key informants.

**Key informant number**	**Organization type**	**Organization level**	**Position level**	**Country**
N1	Government	State/Provincial	Senior	Nigeria
N2	Government	National	Senior	Nigeria
N3	Government	National	Mid-senior	Nigeria
N4	Government	State/Provincial	Senior	Nigeria
N5	Government	State/Provincial	Senior	Nigeria
N6	Government	State/Provincial	Senior	Nigeria
N7	Government	State/Provincial	Senior	Nigeria
N8	Government	National	Mid-senior	Nigeria
N9	NGO	National	Senior	Nigeria
N10	Government	State/Provincial	Senior	Nigeria
N11	Government	National	Senior	Nigeria
I1	NGO	National	Mid-senior	India
I2	Government	State/Provincial	Senior	India
I3	Government	National	Senior	India
I4	Government	National	Mid-senior	India
I5	Government	State/Provincial	Mid-senior	India
I6	NGO	National	Mid-senior	India
I7	Government	District/City	Mid-senior	India
I8	NGO	National	Senior	India
I9	NGO	National	Mid	India
I10	NGO	National	Mid	India
I11	Academia	National	Senior	India
D1	Government	District/City	Mid-level	Indonesia
D2	Government	National	Senior-level	Indonesia
D3	Government	District/City	Mid-level	Indonesia
D4	Government	District/City	Senior-level	Indonesia
D5	NGO	District/City	Mid-level	Indonesia
D6	NGO	State/Provincial	Senior-level	Indonesia
D7	Government	State/Provincial	Senior-level	Indonesia
D8	NGO	State/Provincial	Senior-level	Indonesia
D9	Government	District/City	Mid-level	Indonesia
D10	Government	State/Provincial	Mid-level	Indonesia
D11	NGO	District/City	Senior-level	Indonesia

**Fig. 3:**
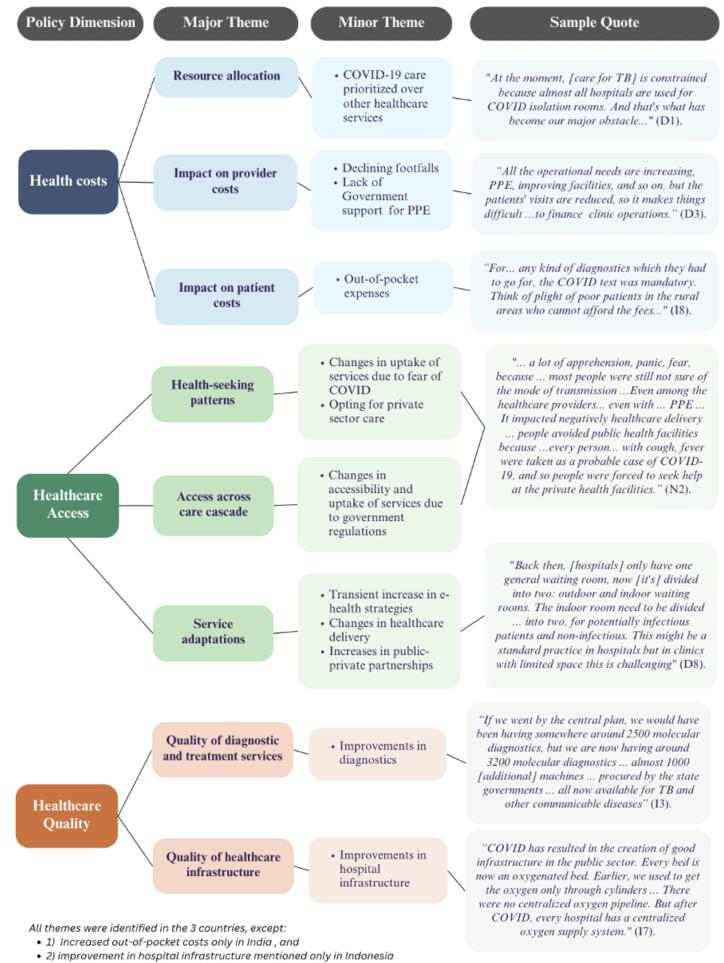
Emerging themes and sample quotes from the analysis.

The emerging themes from the analysis, as well as sample quotes and a comparison of themes across the three countries are presented in Fig. 3.

### 3.1 Healthcare costs

#### 3.1.1 Healthcare resource allocation

Policymakers mentioned that COVID-19 became the priority during the pandemic, with resources reallocated away from TB services. In India, NGOs working through the Patient Provider Support Agency (PPSA) identified local, mid-level hospitals with beds and chest physicians to provide COVID-19 services subsidized by the government. While these subsidies enabled these hospitals to provide life-saving services to individuals affected with COVID-19, while maintaining a steady income throughout the pandemic, the reallocation significantly reduced access to services for TB and other essential health services.

In Indonesia, TB services were hampered by staff diversion to COVID-19 activities, including contact tracing, reporting, and vaccination. As COVID-19 cases increased, the Ministry of Health directed all private hospitals to reallocate TB isolation rooms to treat moderate and severe COVID-19 patients. This particularly affected individuals with drug-resistant TB (DR-TB) requiring hospitalization for severe symptoms and side-effect management *(*Fig. 3 *Health costs, D1).*

In Nigeria, COVID-19 services were centralized to the public sector. While this preserved private sector resources for TB, private facilities had to turn away patients with TB-COVID overlapping symptoms since they couldn't treat COVID-19. As a senior manager put it, “*Eventually, policies that largely restricted management of COVID-19 cases to public facilities were implemented, and healthcare workers from private facilities avoided treating anyone with a cough indicating ‘we are not allowed to manage COVID in this hospital’”* (N9).

COVID-19 was prioritized in all three countries. However in Nigeria, COVID-19 management mainly affected the public sector. This placed constraints on the types of symptoms the private sector could address since patients presenting with COVID-like symptoms could not receive care in a private facility and had to be directed to the public sector. In India and Indonesia, healthcare resources were also funelled to the pandemic which largely affected TB services.

#### 3.1.2 Impact on provider costs

The COVID-19 pandemic led to many hardships for the private sector, with many facilities having to close or reduce staff to cut down operational costs.

In India, private providers faced issues, particularly during the first wave of the pandemic, as patient footfalls drastically reduced. Many private sector hospitals struggled to thrive due to their severely hampered revenue, in addition to having to implement new infection control procedures and procure personal protective equipment (PPE) for staff.

In Indonesia, financial hardship was mitigated for private clinics who established collaborations with the National Health Insurance Scheme (BPJS). Such clinics fared better as they received funds for procurement of PPE and infection control materials, despite experiencing limitations in terms of visiting hours and decreasing number of visits due to the pandemic. “*For private practices…not cooperating with BPJS, they will be more vulnerable; … private health facilities who cooperate with BPJS will receive routine funding through capitation. So they will get monthly income although the patients do not come. But practices who do not cooperate with BPJS will need to rely on fee for services*” (D10).

In Nigeria, private facilities dependence on profits from patient visits, made them particularly vulnerable during the early days of the pandemic when attendance decreased dramatically. Many facilities, particularly smaller ones, had to lay-off staff or close completely due to financial strain. High infection rates among facility staff further reduced community trust and attendance. Although some patients preferred services in the private sector to avoid increased risk of infection in the crowded public sector, this did not compensate for the overall losses, with some private facilities struggling to recover.

All three countries saw a decrease in footfalls in the private sector due to fear of infection, while some realized that they were not well-equipped enough in infection control to cope with the demands of the pandemic. However, in Indonesia, policymakers reported about governmental financial support for provision of PPE in private facilities.

#### 3.1.3 Impact on patient costs

In India, the pandemic placed financial constraints on access to private provider services. Stakeholders in India mentioned significant impacts on patient health-related costs. Although the government implemented price caps, informants shared that many private hospitals only accepted payments in cash during the second wave of the pandemic which represented a barrier to access for patients who could not acess cash. Additionally, it was made mandatory that any person consulting with a private provider should be tested for COVID-19, which increased out-of-pocket expenses substantially.

In Indonesia and Nigeria, informants shared that patient costs for COVID-19 testing were controlled by the government, thus did not vary.

### 3.2 Healthcare access

#### 3.2.1 Health-seeking patterns

Policymakers shared how patient felts felt and apprehension following the onset of the pandemic and the resulting restrictions (Fig. 3 *Healthcare Access N2*). In India, it was mentioned that many were afraid that if they tested positive for COVID-19 they would be placed in quarantine, which resulted in many individuals being hesitant to seek COVID-19 testing. Even patients with chest symptoms hesitated to reveal the symptoms suggestive of TB. One policymaker in India mentioned, “*They were scared that they would be labelled as a COVID patient and be taken for quarantine or be admitted into a hospital. They would hesitate to say that they have a cough when they came … in the fear of getting quarantined or admitted. This is one of the reasons for the delay in the diagnosis of TB*” (I2).

Health-seeking patterns in all three countries were significantly affected by fear of COVID-19 infection. Concerns arose mainly due to the overlap between TB and COVID-19 symptoms. In India, an estimated 70 to 75 % of private clinics were closed as providers feared infection druing outpatient services, creating additional barriers for individuals seeking care. Indonesian public and private healthcare facilities reported significant decreases in patient visits, particularly from patients with respiratory symptoms, for fear of being at higher risk of getting COVID-19, avoided care until their symptoms became severe. In Nigeria, individuals feared being labeled as COVID-19 cases due to overlapping symptoms with TB (cough, fever, weight loss, weakness, or difficulty breathing). Healthcare workers were also more afraid to see patients. However, patients were reported as being more comfortable seeking care in the private sector.

#### 3.2.2 Access across the care cascade

Many individuals faced challenges accessing TB services due to lockdowns and fear of infection. In India, PPSAs helped to mitigate these barriers managing sample collection and transportation, and securing grants to provide treatment for multidrug-resistant TB (MDR-TB). However, laboratory testing of sputum samples using PCR or sputum smear became more difficult to access, as laboratory resources (staff, equipment) were diverted to COVID-19 testing, leaving private facilities to rely primarily on chest x-rays which had an important impact on diagnosis. To mitigate the impact of MDR-TB, which was an important focus for many states, COVID-19 testing transportation services were merged with sputum sample transportation of individuals with MDR-TB in one state. Despite certain efforts in India to maintain treatment, particularly for individuals with DR-TB, this was a challenge due to limited follow-up with patients by either private clinicians or community health workers.

In Indonesia, policymakers noted a decrease in TB testing due to diversion of laboratory resources to COVID-19, as well as reluctance from laboratory staff to test sputum samples for fear of infection in the early days of the pandemic. Across most of the country, to increase access for TB testing, a specimen transport system by a specialized courier called SITRUST (*Sistem Informasi Treking untuk Spesimen Transport*/Tracking Information System for Specimen Transport) was established before the pandemic. As the government implemented massive lockdowns that limited citizens’ mobility, the service was also temporarily halted. When the mobility restriction was lifted, the health office was able to reactivate the system, enabling them to direct the delivery of specimens to available laboratories. “*When there were disruption at the start, the number of patients decrease, we reactivate SITRUST. So when in certain health facilities, they cannot perform lab exam, they can send the specimen to other facilities who still had it going, that was the adjustment we did. And then afterwards when the PPEs were available again, those were carried as normal”* (D1). However, it was also noted that couriers from the service experienced fear of infection when visiting testing sites, especially health facilities with high COVID-19 load. In Indonesia, public health care facilities were overwhelmed with COVID-19 responses such as contact investigation, COVID-19 testing, and vaccination programs. This influenced patients’ perceptions that public health services posed a threat for COVID-19 infection and drove some patients to seek care for their respiratory symptoms at private facilities.

In Nigeria, the pandemic resulted in complete lockdowns in Kano and Lagos during which individuals’ movements were restricted which created challenges in care-seeking. Certain healthcare workers were provided with special passes to deliver healthcare to their patients. Despite this, many individuals with TB experienced diagnostic delays and treatment interruptions, and often they could not access information on where and when they could access treatment during lockdowns. Despite these barriers to access, on informant indicated that there could have been an increase in TB case notification in the private sector, partly because, while public facilities were overwhelmed with COVID-19 cases, many private facilities continued to offer TB services. “*Around that time, …the case notification in the private sector had an improvement of 108 % increment”* (N2).

In Nigeria, efforts were made to maintain access to TB services through intersectoral collaboration. There was close collaboration between private facilities and the government to coordinate COVID-19 screening. Policymakers mentioned that the government also collaborated with NGOs and implementation partners such as SHOPS Plus to implement COVID-19 control measures on the ground, and in some cases to maintain TB case finding. Concerted efforts were also required through coordination of various governmental agencies such as the Private Health Institutions Management Agency (PHIMA) which served as a link to collaborate with the private sector network in the fight against TB. The government was consequently able to engage private laboratories for COVID-19 testing in Kano in previously underserved areas. Policymakers also expressed how the favourable response from the private sector has been a key factor in addressing the pandemic.

In all three countries, policymakers described changes in the uptake and coverage of TB services in the private sector due to fear of infection among patients and healthcare workers, as well as due to implementation of pandemic-related regulations. Additionally, in Indonesia and Nigeria, patients with respiratory symptoms preferentially sought care in the private sector during the pandemic. In Nigeria, intersectoral collaboration was strengthened which resulted in active engagement of the private sector in the fight against TB.

#### 3.2.3 Service adaptations

In India, we noticed an increase in the uptake and use of telemedicine. However, both providers and patients preferred in-person services, particularly in smaller towns where telephone network coverage was not optimal for this kind of service. In India, another adaptation that occurred was flexible service provision which allowed for home visits from doctors, including performing some minor surgeries at home. Certain states integrated TB screening as part of the screening for Influenza-like Illness Severe Acute Respiratory Infections and some states also made wider use of artificial intelligence to read chest x-rays to screen for TB.

In Nigeria, a telemedicine solution was implemented for at-home management of less severe cases of COVID-19. The TB-COVID-19 response team was the main mechanism put in place by the Kano State TB program to address the COVID-19 pandemic and its impact on TB services. Within this program, various sub-committees were organized to address TB services, including diagnostics, treatment, supply chain, and logistics. Another measure that was taken was to restrict management of COVID-19 cases to public facilities. Private facilities provided treatment to mild COVID-19 cases undergoing home-based care under the supervision of the COVID-19 response team. However, management of severe cases was done at accredited facilities. The government placed limits on the number of patients accredited facilities could treat at one time. COVID-19 testing was also limited to accredited facilities. Private facilities which did not have accreditation and were found to be treating COVID-19 cases were shut down. Though the government provided PPE to private facilities, it was reported that the private sector did not receive bailout funds, unlike other healthcare sectors. The Nigerian National TB Programme (NTP) enabled monthly prescription refills for TB which reduced health facility visits for TB patients. In both Nigeria and Indonesia, there was a greater focus on strengthening PPP. In Nigeria, the biggest facilitators in pandemic management was the pre-existing organized structure of the private sector in the country. The government had previously established a link to the private sector through PHIMA which they were able to leverage to provide COVID-19 services outside of the overwhelmed public sector. One key informant referred to the private sector’s contribution as a “*shock absorber*” (N3) as they took on as much as they could. Private facilities were also said to be receptive to infection prevention training they were provided, and they appreciated collaborations with the government.

In Indonesia, policymakers mentioned that there was improved collaboration between public health services, local health office, and private providers and institutions. New interactions were established between the government and professional organizations, and these connections can now be used to discuss other issues, including TB. Professional organizations expressed that the government was more inclined to involve them in policymaking which they considered crucial in achieving common goals. Furthermore, the use of online communication platforms facilitated meetings for diverse organizations which led to better communication.

Policymakers from the 3 countries used telemedicine and policy changes to adapt to the challenges of the pandemic. Additionally, key informants from Nigeria and Indonesia discussed the strengthening of PPP as an important element in adapting to the pandemic.

### 3.3 Healthcare quality

#### 3.3.1 Quality of diagnostic and treatment services

Decreased access to diagnostic services was noted as a challenge by policymakers in all three countries. Although policymakers in our study mentioned that TB diagnosis was affected during the pandemic, as priority was given to COVID-19 testing, all countries did eventually implement systems for sputum transport or the use of alternative screening and diagnostic solutions such as AI. In India, there was an additional concern of shortages due to the shortage of cartridge-based nucleic acid amplification tests in many states, which resulted in COVID-19 testing being prioritized over TB testing. Further, clinicians tended to make diagnoses based on clinical symptoms rather than microbiological testing, since many private diagnostic centers were closed.

#### 3.3.2 Quality of healthcare infrastructure

Despite challenges with access to diagnostics reported in all countries, in India, various policymakers discussed how the pandemic had forced an improvement in healthcare infrastructure across the country. The pandemic resulted in the provision of additional molecular testing machines which can now be made available for TB testing or other communicable diseases.

This improvement in healthcare infrastructure as a result of the pandemic was uniquely reported by key informants in India. Hospitals were provided with equipment such as ventilators that were now used for non-COVID-19 cases. Respondents discussed that the pandemic has resulted in increased knowledge and awareness about outbreak management and infection control. Additionally, pockets of the population with comorbidities were prioritized for testing and vaccination, which increased access to services for these individuals.

### 3.4 Synthesis of health equity policy impacts in the 3 countries

**Fig. 4:**
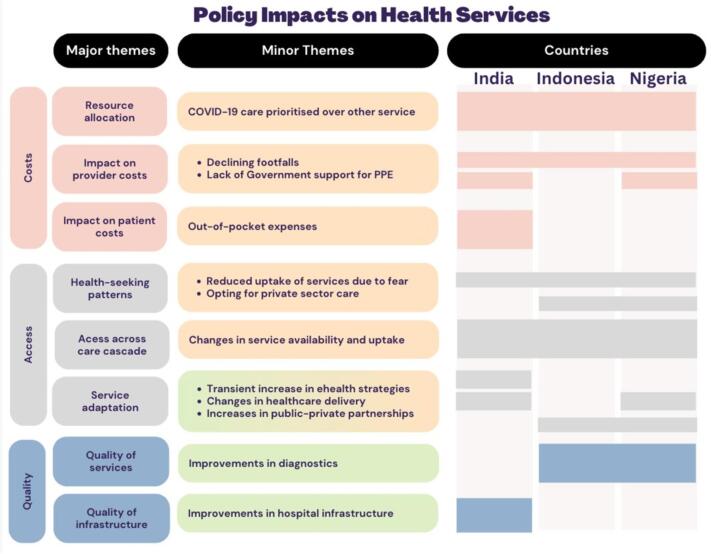
Policy impacts on TB private health services during COVID-19.

In summary (Fig. 4), when we look at the impact of the pandemic on costs, access and quality using the WHO Health Equity Policy framework, there were some similarities and differences between countries.

Related to costs, all countries experienced similar diversion of resources to COVID control and declining patient footfalls, particularly in 2021, with the public healthcare sector taking centre stage in the national governments' COVID-19 response. This left private providers dealing with increasing costs particularly related to decreased client footfalls, providing ‘safe’ care for individuals with TB, including the additional costs of personal protection equipment (PPEs) and building renovations. Only in Indonesia did national governments support private providers for these additional costs. This lack of government support in India and Nigeria negatively impacted the quality of healthcare delivery. We also found catastrophic out-of-pocket expenses for patients in India which would later lead to the government enforcing price caps.

For access, we saw in all countries reduced uptake of services due to fear of COVID-19 infection, with patients in Indonesia and Nigeria opting mostly for private sector care to avoid long wait times in public facilities. There were also reductions in available care across the care cascade (testing, diagnosis and treatment). We saw short-lived increases in the use of e-health and artificial intelligence in India, with a good number of private providers offering teleconsultations with patients. There were notable changes in how care was delivered in India and Nigeria, with private providers offering home-based care for TB and COVID-19. We saw an increase in PPP in Nigeria and Indonesia, with national programs leaning into the services provided by the private sector to ensure continuity of care for patients with TB.

For quality, we saw fewer challenges in accessing diagnostic facilities in Nigeria and Indonesia, as the two countries implemented a combination of strategies among private providers including specialized courier services and case finding for testing and transportation of sputum samples. In India, policymakers mentioned improvement in hospital infrastructure as a direct impact of the national COVID-19 response.

All three countries indicated that although TB diagnosis was affected by the pandemic, the TB services eventually improved. In India, special efforts focused on finding patients with TB lost to follow-up during the pandemic, as well as increased active case finding for TB. In Nigeria, policymakers indicated that cooperation from the private sector dramatically improved compared to before the pandemic, and many months later, infection control protocols were still being followed in private facilities. Many policymakers mentioned the importance of further strengthening engagement with, and increasing capacity building in the private sector. One key informant stated that enhancing collaboration with the private sector beyond the pandemic would strengthen the entire health system:

“So, that it may reach a time, … in which there is no difference, …between … entering into a public or a private health facility in terms of knowledge for the healthcare workers, expertise, as well as services to be rendered because, equipment, … reagents and other logistics consignments will just be provided and at all times… available both in the private health sector as well as the public” (N6).

Some current challenges were mentioned including integrating simultaneous TB and COVID-19 testing, addressing COVID-19 vaccine hesitancy, as well as achieving universal health coverage for all in Nigeria to prevent catastrophic health expenditure, and the role of the private sector in achieving this.

“[…] for us to efficiently and you know, effectively achieve universal health coverage, all hands must be on deck, both private, public, and also the civil society. We must come together if we are serious of achieving universal health coverage by 2030”, (N2).

Our assessment also included the effects of the pandemic-related country-level policies on the equity impacts of effectiveness, coverage, and uptake of TB private sector healthcare.

In understanding effectiveness, all three countries had challenges in distributing resources during COVID-19 which subsequently impacted healthcare provider costs, particularly during the first year of the pandemic. Policymakers mentioned an absence of government support for increased provider costs for additional infection control measures in India and Nigeria. Amidst challenges with laboratory logistics like sputum transportation during lockdown periods, the overall quality of diagnostics and treatment services appeared to improve in all countries as the governments and providers added more diagnostic equipment, provided patient homecare management and became innovative by involving more partners and channels for managing health products. These improvements highlighted the resilience of their healthcare systems, demonstrating abilities to adapt positively in the face of a global health crisis.

Concerning uptake and coverage, the increased costs for providing care likely led to increased consultation costs and catastrophic out-of-pocket expenses, prompting the subsequent implementation of price caps in India. There was a shared challenge of reduced service uptake across all three nations. This decline is attributed to the pervasive fear of COVID-19 infection and alterations in the availability of care throughout the entire care cascade. India, in particular, has witnessed a noteworthy shift in patient preferences toward the public sector. The adaptation strategies employed during the pandemic were evident, including a temporary surge in e-health adoption in India and alterations in service availability in both India and Nigeria. Additionally, Indonesia and Nigeria experienced an encouraging increase in PPP, indicating a collaborative approach to ensure coverage in TB care. Our study underscores the significance of PPP in ensuring the continuity of TB care.

## 4 Discussion

This study provides insights into how COVID-19 impacted private sector TB care delivery in Nigeria, Indonesia and India. Through qualitative analysis of policymaker interviews, we identified themes across three policy dimensions – private sector health costs, healthcare access, and healthcare quality. Our findings highlight both challenges faced by private providers and key adaptations made to maintain TB services during the pandemic.

### 4.1 Health costs

The ‘Covidization’ of health staff and prioritizing COVID-19 during the pandemic led to decreased access to health services. Similar challenges have been highlighted in previous research on the impact of COVID-19. Most recently, a multi-cohort study by Marti et al. found that the pandemic resulted in staff shortages and decreased access to TB care across Asia Pacific and Africa. Disruptions in HIV care were also noted in this study [33]. A previous study by Klinton et al. highlighted the challenges faced by the private sector in seven high-burden TB countries. The authors report that the private sector suffered various financial repercussions from the pandemic in the form of closures due to fear of infection, restrictions, and unexpected expenses [19]. These factors were also highlighted in our study. However, Klinton et al. mention that this led to increased costs for private-sector patients. In the current study, this was only highlighted in India, while Nigeria and Indonesia policymakers did not mention increased out-of-pocket expenses for patients.

### 4.2 Healthcare access

Fear emerged as a critical barrier to TB care in all three countries, with both patients and providers avoiding health services due to COVID-19 infection concerns and overlapping TB-COVID symptoms. This aligns with research in Kenya showing that people with TB felt shame because of overlapping symptoms with COVID-19 [34]. Fear of infection among healthcare workers has also been shown to affect healthcare workers' psychological well-being and the quality of care they provide, highlighting the need for targeted education campaigns and healthcare worker support programs [35].

The private sector demonstrated varying levels of resilience and adaptations during the pandemic in all three countries. In Nigeria, the private sector served as a “shock absorber” when public facilities were overwhelmed with COVID-19 response, contributing to a 50 % increase in TB notifications in 2021 compared to 2020 [36], contrasting with India and Indonesia notification declines due to their higher COVID-19 burden. It is also important to consider that Nigeria’s increased involvement of the private sector in their pandemic response may have played a role in their success. While Indonesia faced significant TB service disruptions particularly during the Delta strain wave [37], [38], [39], [40], adaptations like telemedicine facilitated healthcare access to health services [41]. Private sector services have since returned to and surpassed pre-pandemic levels [42], suggesting the importance of strengthening PPP for future emergency preparedness.

### 4.3 Healthcare quality

The pandemic precipitated some advancements in healthcare quality. Heightened infection control knowledge and awareness triggered a strategic focus on testing and treatment services for vulnerable populations, exemplifying the healthcare system's adaptability. The need for, and the implementation of healthcare adaptations to the pandemic using concerted efforts to improve services have been reported in other studies [19], [43], [44], [45]. Diagnostic improvements in Indonesia and Nigeria, coupled with improved hospital infrastructure in India, underscored a concerted effort towards improving healthcare quality, similar to other studies [45], [46]. The multifaceted strategies employed in coordinating TB care during the pandemic, such as Indonesia leveraging its laboratory network and Nigeria establishing a formal integration structure for TB and COVID-19, showcased a commitment to maintaining quality services. The private sector's pivotal role in delivering TB care amid uncertainty highlighted its contribution to sustaining healthcare quality [19], [44], [46]. Intersectoral collaborations witnessed through initiatives like PHIMA in Nigeria, professional associations (Asklin, ARSSI) in Indonesia, and collaboration with Private Provider Support Agencies (PPSAs) in India, showed ongoing opportunities for enhancing healthcare quality across the three countries.

### 4.4 Recommendations

Based on our analysis of policymaker interviews and experiences, five key recommendations emerged. First, public–private partnerships should be formalized through clear emergency protocols and resource-sharing mechanisms, with sustained collaborative efforts aimed at achieving universal health coverage and preventing catastrophic health costs. Second, TB diagnostic systems need strengthening to address ongoing challenges in case-finding and prevent loss to follow-up, particularly during health emergencies. Third, documented adaptations from COVID-19 should inform protocols for private sector service continuity and health system resilience during future crises. Fourth, robust communication channels between public and private sectors must be established to ensure coordinated emergency response. Fifth, human resource capacity should be built through emergency preparedness training and staff protection protocols. These recommendations aim to enhance health system resilience while maintaining essential TB services during future health emergencies.

### 4.5 Strengths and limitations

A key strength of the current study is its capture of policymaker perspectives in three high TB burden countries, providing insights through a policy lens on PPP during the pandemic. While focused on the private sector, many interviewed policymakers had oversight of both public and private facilities, enabling comparison of policy impacts across sectors. Limitations include reliance on virtual interviews, potential recall bias, and geographical constraints. Our study has two additional important limitations: differences in governance and health system structures affect cross-country comparability, and our reliance on policymakers' perspectives, while informative, may reflect assumptions or biases about patient experiences. Future studies should examine perspectives of healthcare workers, community workers, and patients whose voices are needed to truly understand care-seeking barriers.

## 5 Conclusion

This study examined COVID-19′s impact on private sector TB services through qualitative interviews with policymakers in three high TB burden countries. We found that while the pandemic significantly disrupted TB services through resource diversion and access barriers, it also catalyzed important health system adaptations and strengthened public–private collaboration. Based on these findings, we recommend formalizing public–private partnerships, strengthening TB diagnostic capacity, establishing emergency response protocols, and building robust communication channels between sectors. These evidence-based recommendations aim to enhance health system resilience and maintain essential TB services during future health emergencies.

## Ethical statement

Ethics approval for this study was provided by the following institutions: This study was approved by the McGill University Health Centre (MUHC) Research Ethics Board (REB) (COVID BMGF / 2021–7197), and the Research Ethics Committee of Universitas Padjadjaran, Bandung, Indonesia (No.166/UN6.KEP/EC/2021). In Nigeria, we received approval from the Health Research Ethics Committee (HREC) from the Lagos State University Teaching Hospital (LREC/06/10/1517) and Kano State Ministry of Health (MoH/Off/797/T.1/2168). In India, we received approval from the Institutional Review Board of the Institute for Socio-Economic Research on Development and Democracy (ISERDD/150221).

## CRediT authorship contribution statement

**Nathaly Aguilera Vasquez:** Writing – review & editing, Writing – original draft, Validation, Project administration, Methodology, Investigation, Formal analysis, Data curation, Conceptualization. **Charity Oga-Omenka:** Writing – review & editing, Writing – original draft, Visualization, Validation, Supervision, Project administration, Methodology, Investigation, Formal analysis, Conceptualization. **Vijayashree Yellappa:** Writing – review & editing, Validation, Supervision, Project administration, Methodology, Investigation, Formal analysis, Data curation, Conceptualization. **Bony Wiem Lestari:** Writing – review & editing, Validation, Supervision, Resources, Project administration, Investigation, Formal analysis. **Angelina Sassi:** Writing – review & editing, Validation, Software, Methodology, Investigation, Formal analysis, Conceptualization. **Surbhi Sheokand:** Writing – review & editing, Validation, Project administration, Methodology. **Bolanle Olusola-Faleye:** Writing – review & editing, Validation, Supervision, Resources, Project administration, Investigation. **Lavanya Huria:** Writing – review & editing, Software, Methodology, Formal analysis, Conceptualization. **Laura Jane Brubacher:** Writing – review & editing, Validation, Formal analysis. **Elaine Baruwa:** Writing – review & editing, Supervision, Resources, Investigation, Funding acquisition. **Bachti Alisjahbana:** Writing – review & editing, Supervision, Project administration, Investigation, Funding acquisition. **Madhukar Pai:** Writing – review & editing, Supervision, Resources, Investigation, Funding acquisition, Conceptualization.

## Funding

Funding was provided by the Bill and Melinda Gates Foundation (Grant #: INV-022420) and the University of Waterloo.

## Declaration of competing interest

The authors declare that they have no known competing financial interests or personal relationships that could have appeared to influence the work reported in this paper.
